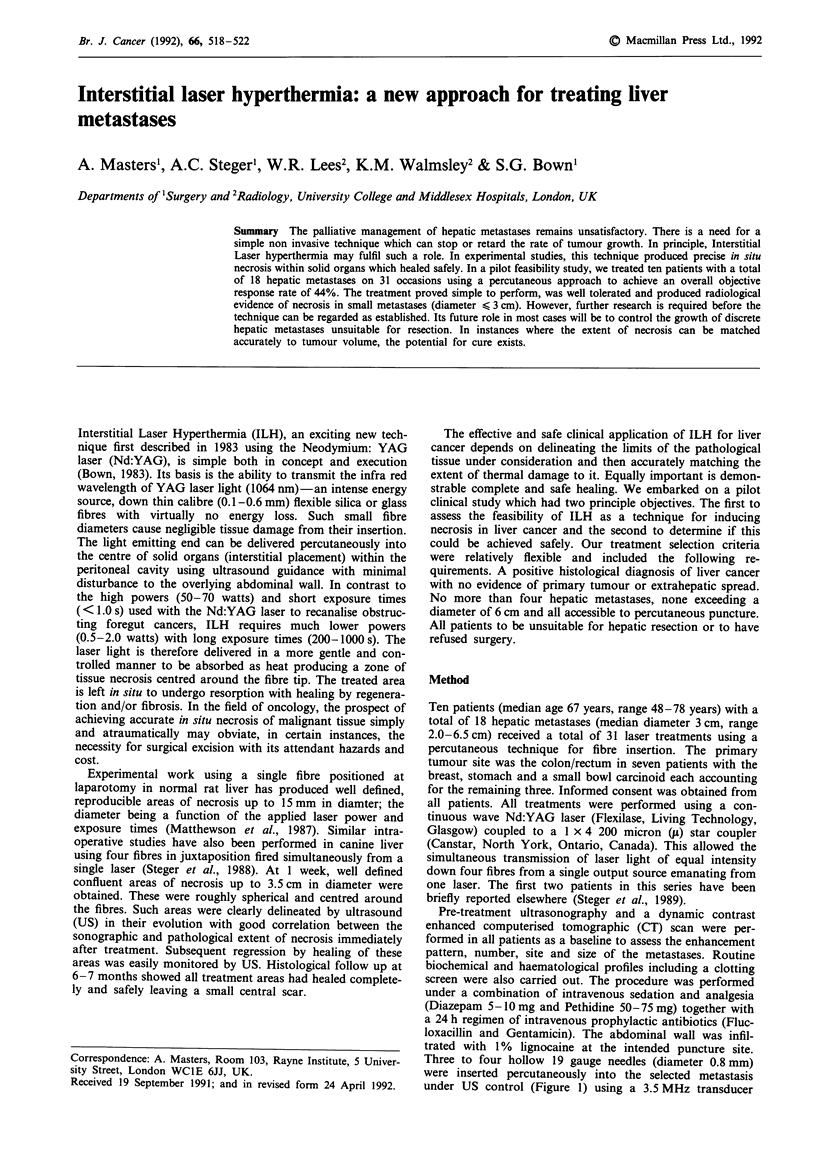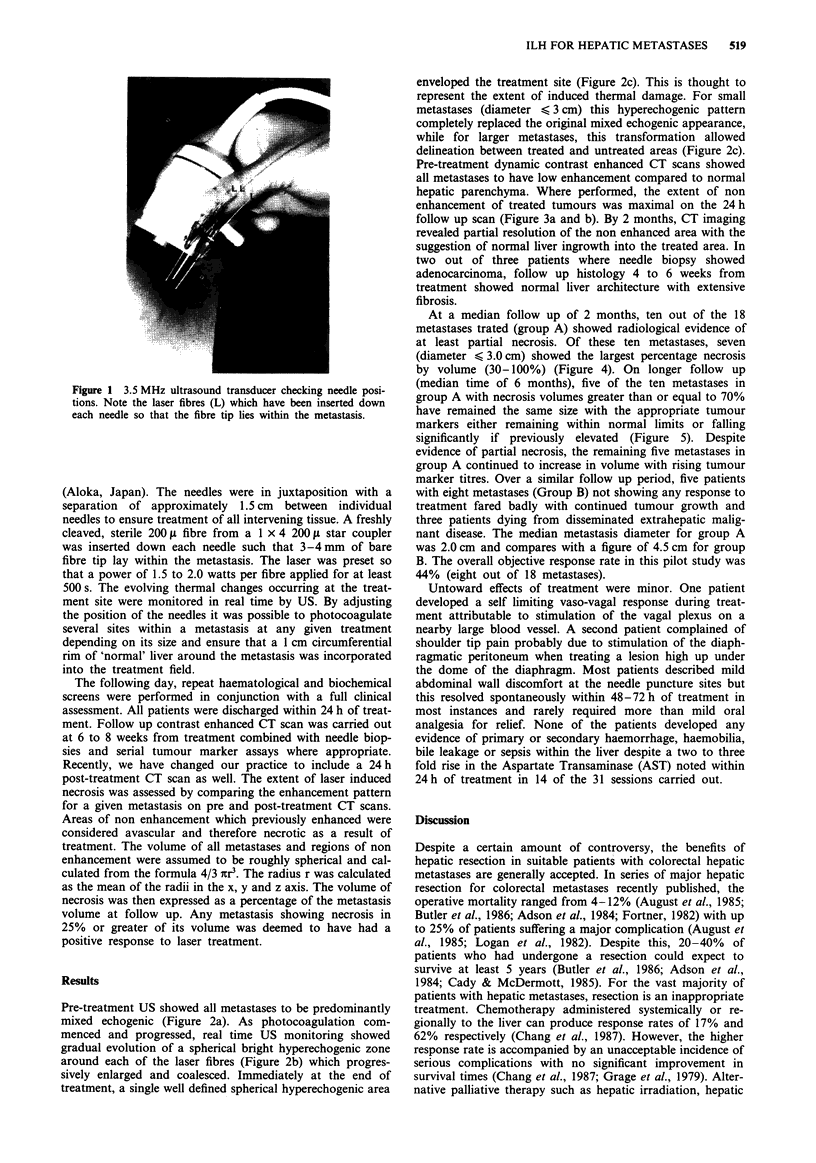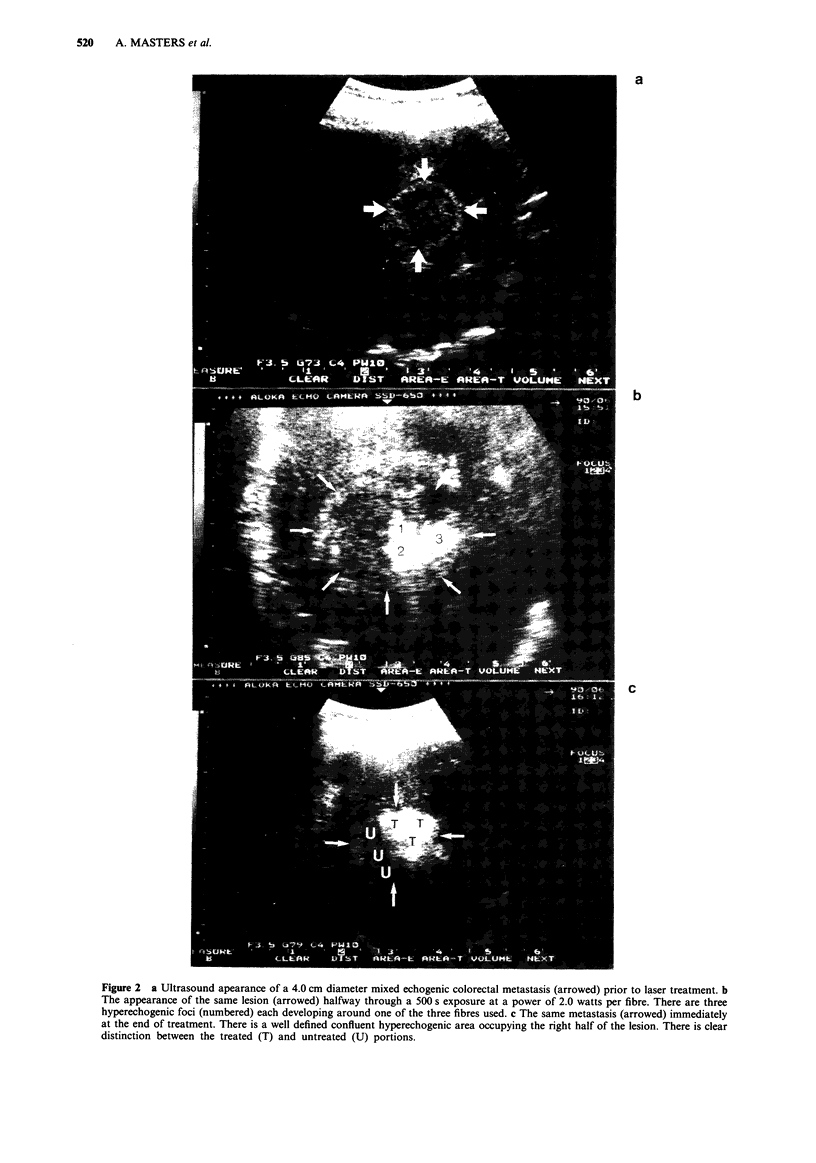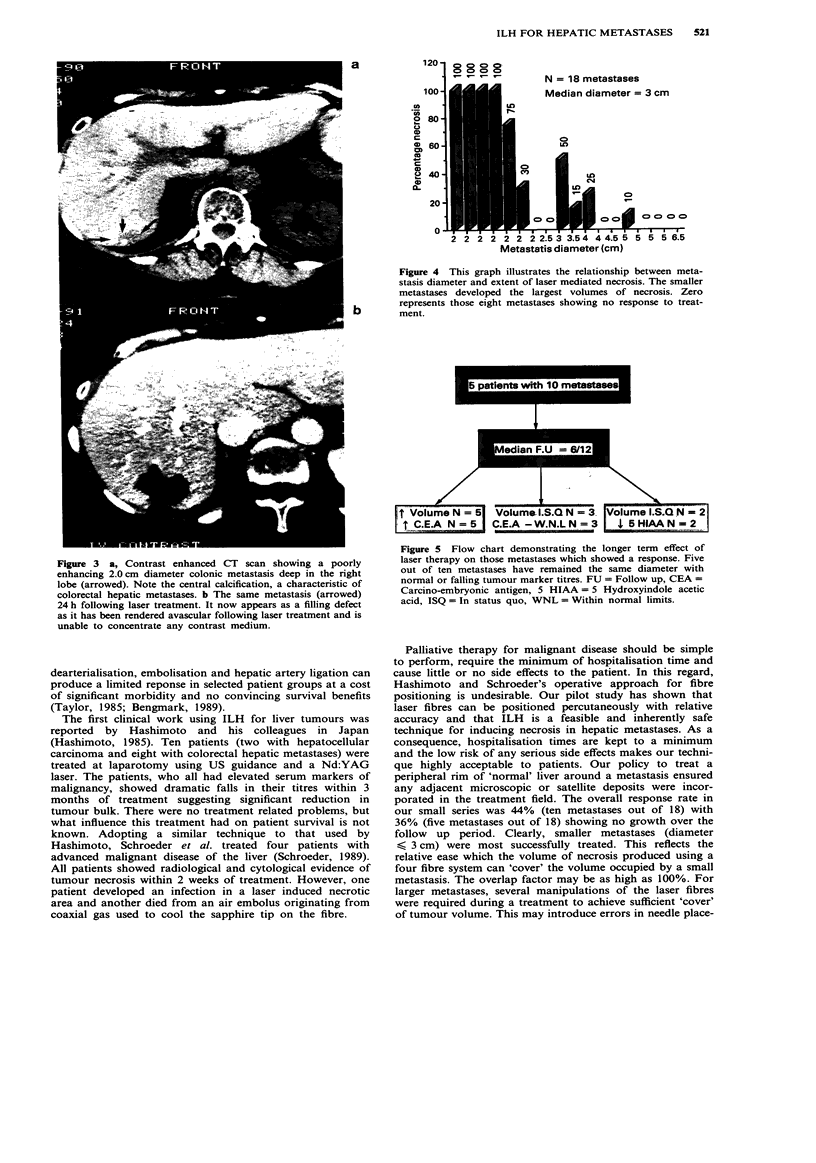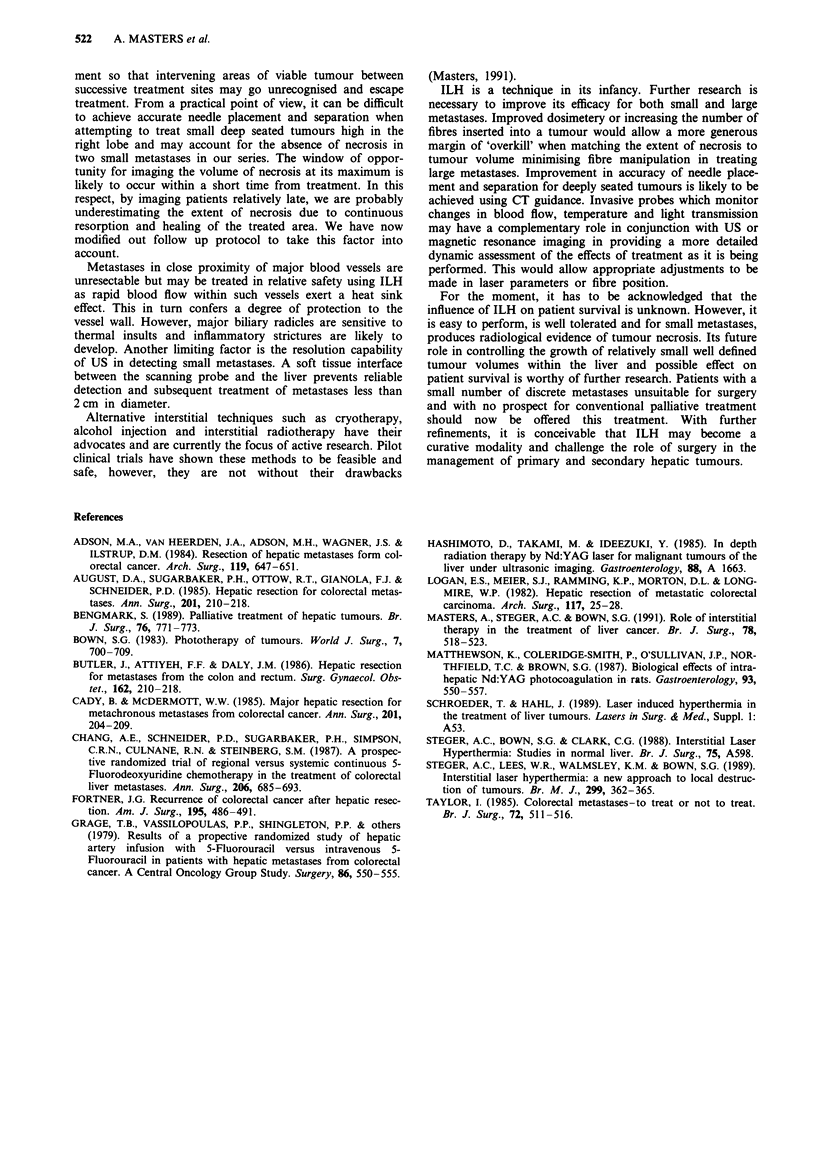# Interstitial laser hyperthermia: a new approach for treating liver metastases.

**DOI:** 10.1038/bjc.1992.305

**Published:** 1992-09

**Authors:** A. Masters, A. C. Steger, W. R. Lees, K. M. Walmsley, S. G. Bown

**Affiliations:** Department of Surgery, University College and Middlesex Hospitals, London, UK.

## Abstract

**Images:**


Br. J. Cancer (1992), 66, 518-522 © Macmillan Press Ltd., 1992

### Interstitial laser hyperthermia: a new approach for treating liver metastases

A. Masters[1], A.C. Steger[1], W.R. Lees[2], K.M. Walmsley[2] & S.G. Bown[1]

*Departments of [1]Surgery and [2]Radiology, University College and Middlesex Hospitals, London, UK*

**Summary** The palliative management of hepatic metastases remains unsatisfactory. There is a need for a simple non invasive technique which can stop or retard the rate of tumour growth. In principle, Interstitial Laser hyperthermia may fulfil such a role. In experimental studies, this technique produced precise *in situ* necrosis within solid organs which healed safely. In a pilot feasibility study, we treated ten patients with a total of 18 hepatic metastases on 31 occasions using a percutaneous approach to achieve an overall objective response rate of 44%. The treatment proved simple to perform, was well tolerated and produced radiological evidence of necrosis in small metastases (diameter ⩽ 3 cm). However, further research is required before the technique can be regarded as established. Its future role in most cases will be to control the growth of discrete hepatic metastases unsuitable for resection. In instances where the extent of necrosis can be matched accurately to tumour volume, the potential for cure exists.

Interstitial Laser Hyperthermia (ILH), an exciting new technique first described in 1983 using the Neodymium: YAG laser (Nd:YAG), is simple both in concept and execution (Bown, 1983). Its basis is the ability to transmit the infra red wavelength of YAG laser light (1064 nm)—an intense energy source, down thin calibre (0.1-0.6 mm) flexible silica or glass fibres with virtually no energy loss. Such small fibre diameters cause negligible tissue damage from their insertion. The light emitting end can be delivered percutaneously into the centre of solid organs (interstitial placement) within the peritoneal cavity using ultrasound guidance with minimal disturbance to the overlying abdominal wall. In contrast to the high powers (50-70 watts) and short exposure times (< 1.0 s) used with the Nd:YAG laser to recanalise obstructing foregut cancers, ILH requires much lower powers (0.5-2.0 watts) with long exposure times (200-1000 s). The laser light is therefore delivered in a more gentle and controlled manner to be absorbed as heat producing a zone of tissue necrosis centred around the fibre tip. The treated area is left *in situ* to undergo resorption with healing by regeneration and/or fibrosis. In the field of oncology, the prospect of achieving accurate *in situ* necrosis of malignant tissue simply and atraumatically may obviate, in certain instances, the necessity for surgical excision with its attendant hazards and cost.

Experimental work using a single fibre positioned at laparotomy in normal rat liver has produced well defined, reproducible areas of necrosis up to 15 mm in diamter; the diameter being a function of the applied laser power and exposure times (Matthewson *et al.*, 1987). Similar intraoperative studies have also been performed in canine liver using four fibres in juxtaposition fired simultaneously from a single laser (Steger *et al.*, 1988). At 1 week, well defined confluent areas of necrosis up to 3.5 cm in diameter were obtained. These were roughly spherical and centred around the fibres. Such areas were clearly delineated by ultrasound (US) in their evolution with good correlation between the sonographic and pathological extent of necrosis immediately after treatment. Subsequent regression by healing of these areas was easily monitored by US. Histological follow up at 6-7 months showed all treatment areas had healed completely and safely leaving a small central scar.

Correspondence: A. Masters, Room 103, Rayne Institute, 5 University Street, London WC1E 6JJ, UK.
Received 19 September 1991; and in revised form 24 April 1992.

The effective and safe clinical application of ILH for liver cancer depends on delineating the limits of the pathological tissue under consideration and then accurately matching the extent of thermal damage to it. Equally important is demonstrable complete and safe healing. We embarked on a pilot clinical study which had two principle objectives. The first to assess the feasibility of ILH as a technique for inducing necrosis in liver cancer and the second to determine if this could be achieved safely. Our treatment selection criteria were relatively flexible and included the following requirements. A positive histological diagnosis of liver cancer with no evidence of primary tumour or extrahepatic spread. No more than four hepatic metastases, none exceeding a diameter of 6 cm and all accessible to percutaneous puncture. All patients to be unsuitable for hepatic resection or to have refused surgery.

### Method

Ten patients (median age 67 years, range 48-78 years) with a total of 18 hepatic metastases (median diameter 3 cm, range 2.0-6.5 cm) received a total of 31 laser treatments using a percutaneous technique for fibre insertion. The primary tumour site was the colon/rectum in seven patients with the breast, stomach and a small bowl carcinoid each accounting for the remaining three. Informed consent was obtained from all patients. All treatments were performed using a continuous wave Nd:YAG laser (Flexilase, Living Technology, Glasgow) coupled to a 1 × 4 200 micron (μ) star coupler (Canstar, North York, Ontario, Canada). This allowed the simultaneous transmission of laser light of equal intensity down four fibres from a single output source emanating from one laser. The first two patients in this series have been briefly reported elsewhere (Steger *et al.*, 1989).

Pre-treatment ultrasonography and a dynamic contrast enhanced computerised tomographic (CT) scan were performed in all patients as a baseline to assess the enhancement pattern, number, site and size of the metastases. Routine biochemical and haematological profiles including a clotting screen were also carried out. The procedure was performed under a combination of intravenous sedation and analgesia (Diazepam 5-10 mg and Pethidine 50-75 mg) together with a 24 h regimen of intravenous prophylactic antibiotics (Flucloxacillin and Gentamicin). The abdominal wall was infiltrated with 1% lignocaine at the intended puncture site. Three to four hollow 19 gauge needles (diameter 0.8 mm) were inserted percutaneously into the selected metastasis under US control (Figure 1) using a 3.5 MHz transducer

**Figure 1** 3.5 MHz ultrasound transducer checking needle positions. Note the laser fibres (L) which have been inserted down each needle so that the fibre tip lies within the metastasis.

(Aloka, Japan). The needles were in juxtaposition with a separation of approximately 1.5 cm between individual needles to ensure treatment of all intervening tissue. A freshly cleaved, sterile 200 μ fibre from a 1 × 4 200 μ star coupler was inserted down each needle such that 3–4 mm of bare fibre tip lay within the metastasis. The laser was preset so that a power of 1.5 to 2.0 watts per fibre applied for at least 500 s. The evolving thermal changes occurring at the treatment site were monitored in real time by US. By adjusting the position of the needles it was possible to photocoagulate several sites within a metastasis at any given treatment depending on its size and ensure that a 1 cm circumferential rim of 'normal' liver around the metastasis was incorporated into the treatment field.

The following day, repeat haematological and biochemical screens were performed in conjunction with a full clinical assessment. All patients were discharged within 24 h of treatment. Follow up contrast enhanced CT scan was carried out at 6 to 8 weeks from treatment combined with needle biopsies and serial tumour marker assays where appropriate. Recently, we have changed our practice to include a 24 h post-treatment CT scan as well. The extent of laser induced necrosis was assessed by comparing the enhancement pattern for a given metastasis on pre and post-treatment CT scans. Areas of non enhancement which previously enhanced were considered avascular and therefore necrotic as a result of treatment. The volume of all metastases and regions of non enhancement were assumed to be roughly spherical and calculated from the formula $4/3\ \pi r^3$. The radius r was calculated as the mean of the radii in the x, y and z axis. The volume of necrosis was then expressed as a percentage of the metastasis volume at follow up. Any metastasis showing necrosis in 25% or greater of its volume was deemed to have had a positive response to laser treatment.

### Results

Pre-treatment US showed all metastases to be predominantly mixed echogenic (Figure 2a). As photocoagulation commenced and progressed, real time US monitoring showed gradual evolution of a spherical bright hyperechogenic zone around each of the laser fibres (Figure 2b) which progressively enlarged and coalesced. Immediately at the end of treatment, a single well defined spherical hyperechogenic area enveloped the treatment site (Figure 2c). This is thought to represent the extent of induced thermal damage. For small metastases (diameter $\leqslant$ 3 cm) this hyperechogenic pattern completely replaced the original mixed echogenic appearance, while for larger metastases, this transformation allowed delineation between treated and untreated areas (Figure 2c). Pre-treatment dynamic contrast enhanced CT scans showed all metastases to have low enhancement compared to normal hepatic parenchyma. Where performed, the extent of non enhancement of treated tumours was maximal on the 24 h follow up scan (Figure 3a and b). By 2 months, CT imaging revealed partial resolution of the non enhanced area with the suggestion of normal liver ingrowth into the treated area. In two out of three patients where needle biopsy showed adenocarcinoma, follow up histology 4 to 6 weeks from treatment showed normal liver architecture with extensive fibrosis.

At a median follow up of 2 months, ten out of the 18 metastases trated (group A) showed radiological evidence of at least partial necrosis. Of these ten metastases, seven (diameter $\leqslant$ 3.0 cm) showed the largest percentage necrosis by volume (30–100%) (Figure 4). On longer follow up (median time of 6 months), five of the ten metastases in group A with necrosis volumes greater than or equal to 70% have remained the same size with the appropriate tumour markers either remaining within normal limits or falling significantly if previously elevated (Figure 5). Despite evidence of partial necrosis, the remaining five metastases in group A continued to increase in volume with rising tumour marker titres. Over a similar follow up period, five patients with eight metastases (Group B) not showing any response to treatment fared badly with continued tumour growth and three patients dying from disseminated extrahepatic malignant disease. The median metastasis diameter for group A was 2.0 cm and compares with a figure of 4.5 cm for group B. The overall objective response rate in this pilot study was 44% (eight out of 18 metastases).

Untoward effects of treatment were minor. One patient developed a self limiting vaso-vagal response during treatment attributable to stimulation of the vagal plexus on a nearby large blood vessel. A second patient complained of shoulder tip pain probably due to stimulation of the diaphragmatic peritoneum when treating a lesion high up under the dome of the diaphragm. Most patients described mild abdominal wall discomfort at the needle puncture sites but this resolved spontaneously within 48–72 h of treatment in most instances and rarely required more than mild oral analgesia for relief. None of the patients developed any evidence of primary or secondary haemorrhage, haemobilia, bile leakage or sepsis within the liver despite a two to three fold rise in the Aspartate Transaminase (AST) noted within 24 h of treatment in 14 of the 31 sessions carried out.

### Discussion

Despite a certain amount of controversy, the benefits of hepatic resection in suitable patients with colorectal hepatic metastases are generally accepted. In series of major hepatic resection for colorectal metastases recently published, the operative mortality ranged from 4–12% (August *et al.*, 1985; Butler *et al.*, 1986; Adson *et al.*, 1984; Fortner, 1982) with up to 25% of patients suffering a major complication (August *et al.*, 1985; Logan *et al.*, 1982). Despite this, 20–40% of patients who had undergone a resection could expect to survive at least 5 years (Butler *et al.*, 1986; Adson *et al.*, 1984; Cady & McDermott, 1985). For the vast majority of patients with hepatic metastases, resection is an inappropriate treatment. Chemotherapy administered systemically or regionally to the liver can produce response rates of 17% and 62% respectively (Chang *et al.*, 1987). However, the higher response rate is accompanied by an unacceptable incidence of serious complications with no significant improvement in survival times (Chang *et al.*, 1987; Grage *et al.*, 1979). Alternative palliative therapy such as hepatic irradiation, hepatic

**Figure 2** **a** Ultrasound apearance of a 4.0 cm diameter mixed echogenic colorectal metastasis (arrowed) prior to laser treatment. **b** The appearance of the same lesion (arrowed) halfway through a 500 s exposure at a power of 2.0 watts per fibre. There are three hyperechogenic foci (numbered) each developing around one of the three fibres used. **c** The same metastasis (arrowed) immediately at the end of treatment. There is a well defined confluent hyperechogenic area occupying the right half of the lesion. There is clear distinction between the treated (T) and untreated (U) portions.

**Figure 3** **a,** Contrast enhanced CT scan showing a poorly enhancing 2.0 cm diameter colonic metastasis deep in the right lobe (arrowed). Note the central calcification, a characteristic of colorectal hepatic metastases. **b** The same metastasis (arrowed) 24 h following laser treatment. It now appears as a filling defect as it has been rendered avascular following laser treatment and is unable to concentrate any contrast medium.

**Figure 4** This graph illustrates the relationship between metastasis diameter and extent of laser mediated necrosis. The smaller metastases developed the largest volumes of necrosis. Zero represents those eight metastases showing no response to treatment.

**Figure 5** Flow chart demonstrating the longer term effect of laser therapy on those metastases which showed a response. Five out of ten metastases have remained the same diameter with normal or falling tumour marker titres. FU = Follow up, CEA = Carcino-embryonic antigen, 5 HIAA = 5 Hydroxyindole acetic acid, ISQ = In status quo, WNL = Within normal limits.

dearterialisation, embolisation and hepatic artery ligation can produce a limited reponse in selected patient groups at a cost of significant morbidity and no convincing survival benefits (Taylor, 1985; Bengmark, 1989).

The first clinical work using ILH for liver tumours was reported by Hashimoto and his colleagues in Japan (Hashimoto, 1985). Ten patients (two with hepatocellular carcinoma and eight with colorectal hepatic metastases) were treated at laparotomy using US guidance and a Nd:YAG laser. The patients, who all had elevated serum markers of malignancy, showed dramatic falls in their titres within 3 months of treatment suggesting significant reduction in tumour bulk. There were no treatment related problems, but what influence this treatment had on patient survival is not known. Adopting a similar technique to that used by Hashimoto, Schroeder *et al.* treated four patients with advanced malignant disease of the liver (Schroeder, 1989). All patients showed radiological and cytological evidence of tumour necrosis within 2 weeks of treatment. However, one patient developed an infection in a laser induced necrotic area and another died from an air embolus originating from coaxial gas used to cool the sapphire tip on the fibre.

Palliative therapy for malignant disease should be simple to perform, require the minimum of hospitalisation time and cause little or no side effects to the patient. In this regard, Hashimoto and Schroeder's operative approach for fibre positioning is undesirable. Our pilot study has shown that laser fibres can be positioned percutaneously with relative accuracy and that ILH is a feasible and inherently safe technique for inducing necrosis in hepatic metastases. As a consequence, hospitalisation times are kept to a minimum and the low risk of any serious side effects makes our technique highly acceptable to patients. Our policy to treat a peripheral rim of 'normal' liver around a metastasis ensured any adjacent microscopic or satellite deposits were incorporated in the treatment field. The overall response rate in our small series was 44% (ten metastases out of 18) with 36% (five metastases out of 18) showing no growth over the follow up period. Clearly, smaller metastases (diameter $\leqslant$ 3 cm) were most successfully treated. This reflects the relative ease which the volume of necrosis produced using a four fibre system can 'cover' the volume occupied by a small metastasis. The overlap factor may be as high as 100%. For larger metastases, several manipulations of the laser fibres were required during a treatment to achieve sufficient 'cover' of tumour volume. This may introduce errors in needle placement so that intervening areas of viable tumour between successive treatment sites may go unrecognised and escape treatment. From a practical point of view, it can be difficult to achieve accurate needle placement and separation when attempting to treat small deep seated tumours high in the right lobe and may account for the absence of necrosis in two small metastases in our series. The window of opportunity for imaging the volume of necrosis at its maximum is likely to occur within a short time from treatment. In this respect, by imaging patients relatively late, we are probably underestimating the extent of necrosis due to continuous resorption and healing of the treated area. We have now modified out follow up protocol to take this factor into account.

Metastases in close proximity of major blood vessels are unresectable but may be treated in relative safety using ILH as rapid blood flow within such vessels exert a heat sink effect. This in turn confers a degree of protection to the vessel wall. However, major biliary radicles are sensitive to thermal insults and inflammatory strictures are likely to develop. Another limiting factor is the resolution capability of US in detecting small metastases. A soft tissue interface between the scanning probe and the liver prevents reliable detection and subsequent treatment of metastases less than 2 cm in diameter.

Alternative interstitial techniques such as cryotherapy, alcohol injection and interstitial radiotherapy have their advocates and are currently the focus of active research. Pilot clinical trials have shown these methods to be feasible and safe, however, they are not without their drawbacks (Masters, 1991).

ILH is a technique in its infancy. Further research is necessary to improve its efficacy for both small and large metastases. Improved dosimetery or increasing the number of fibres inserted into a tumour would allow a more generous margin of 'overkill' when matching the extent of necrosis to tumour volume minimising fibre manipulation in treating large metastases. Improvement in accuracy of needle placement and separation for deeply seated tumours is likely to be achieved using CT guidance. Invasive probes which monitor changes in blood flow, temperature and light transmission may have a complementary role in conjunction with US or magnetic resonance imaging in providing a more detailed dynamic assessment of the effects of treatment as it is being performed. This would allow appropriate adjustments to be made in laser parameters or fibre position.

For the moment, it has to be acknowledged that the influence of ILH on patient survival is unknown. However, it is easy to perform, is well tolerated and for small metastases, produces radiological evidence of tumour necrosis. Its future role in controlling the growth of relatively small well defined tumour volumes within the liver and possible effect on patient survival is worthy of further research. Patients with a small number of discrete metastases unsuitable for surgery and with no prospect for conventional palliative treatment should now be offered this treatment. With further refinements, it is conceivable that ILH may become a curative modality and challenge the role of surgery in the management of primary and secondary hepatic tumours.

### References

ADSON, M.A., VAN HEERDEN, J.A., ADSON, M.H., WAGNER, J.S. & ILSTRUP, D.M. (1984). Resection of hepatic metastases form colorectal cancer. *Arch. Surg.*, **119**, 647-651.

AUGUST, D.A., SUGARBAKER, P.H., OTTOW, R.T., GIANOLA, F.J. & SCHNEIDER, P.D. (1985). Hepatic resection for colorectal metastases. *Ann. Surg.*, **201**, 210-218.

BENGMARK, S. (1989). Palliative treatment of hepatic tumours. *Br. J. Surg.*, **76**, 771-773.

BOWN, S.G. (1983). Phototherapy of tumours. *World J. Surg.*, **7**, 700-709.

BUTLER, J., ATTIYEH, F.F. & DALY, J.M. (1986). Hepatic resection for metastases from the colon and rectum. *Surg. Gynaecol. Obstet.*, **162**, 210-218.

CADY, B. & MCDERMOTT, W.W. (1985). Major hepatic resection for metachronous metastases from colorectal cancer. *Ann. Surg.*, **201**, 204-209.

CHANG, A.E., SCHNEIDER, P.D., SUGARBAKER, P.H., SIMPSON, C.R.N., CULNANE, R.N. & STEINBERG, S.M. (1987). A prospective randomized trial of regional versus systemic continuous 5-Fluorodeoxyuridine chemotherapy in the treatment of colorectal liver metastases. *Ann. Surg.*, **206**, 685-693.

FORTNER, J.G. Recurrence of colorectal cancer after hepatic resection. *Am. J. Surg.*, **195**, 486-491.

GRAGE, T.B., VASSILOPOULAS, P.P., SHINGLETON, P.P. & others (1979). Results of a propective randomized study of hepatic artery infusion with 5-Fluorouracil versus intravenous 5-Fluorouracil in patients with hepatic metastases from colorectal cancer. A Central Oncology Group Study. *Surgery*, **86**, 550-555.

HASHIMOTO, D., TAKAMI, M. & IDEEZUKI, Y. (1985). In depth radiation therapy by Nd:YAG laser for malignant tumours of the liver under ultrasonic imaging. *Gastroenterology*, **88**, A 1663.

LOGAN, E.S., MEIER, S.J., RAMMING, K.P., MORTON, D.L. & LONGMIRE, W.P. (1982). Hepatic resection of metastatic colorectal carcinoma. *Arch. Surg.*, **117**, 25-28.

MASTERS, A., STEGER, A.C. & BOWN, S.G. (1991). Role of interstitial therapy in the treatment of liver cancer. *Br. J. Surg.*, **78**, 518-523.

MATTHEWSON, K., COLERIDGE-SMITH, P., O'SULLIVAN, J.P., NORTHFIELD, T.C. & BROWN, S.G. (1987). Biological effects of intrahepatic Nd:YAG photocoagulation in rats. *Gastroenterology*, **93**, 550-557.

SCHROEDER, T. & HAHL, J. (1989). Laser induced hyperthermia in the treatment of liver tumours. *Lasers in Surg. & Med.*, Suppl. 1: A53.

STEGER, A.C., BOWN, S.G. & CLARK, C.G. (1988). Interstitial Laser Hyperthermia: Studies in normal liver. *Br. J. Surg.*, **75**, A598.

STEGER, A.C., LEES, W.R., WALMSLEY, K.M. & BOWN, S.G. (1989). Interstitial laser hyperthermia: a new approach to local destruction of tumours. *Br. M. J.*, **299**, 362-365.

TAYLOR, I. (1985). Colorectal metastases-to treat or not to treat. *Br. J. Surg.*, **72**, 511-516.